# PPAR-γ promotes p38 MAP kinase-mediated endothelial cell permeability through activating Sirt3

**DOI:** 10.1186/s12883-019-1508-y

**Published:** 2019-11-15

**Authors:** Zhenzhen Zhao, Xiaoxiu Zhang, Yuanqiang Dai, Ke Pan, Yu Deng, Yan Meng, Tao Xu

**Affiliations:** Department of Anesthesiology, Changhai hospital, Naval Medical University, Changhai Road NO.168, Shanghai City, 200433 People’s Republic of China

**Keywords:** PPAR-γ, p38, Sirt3, Cell permeability, Ischemia-reperfusion

## Abstract

**Background:**

Ischemia-reperfusion (I/R)-induced vascular dysfunction is the main factor to acute ischemic stroke. Sirt3 is one of the sirtuin family members, which plays an important role in the development of neurological diseases.

**Methods:**

In this study, we constructed I/R injury model on HBMEC cells and induced the overexpression of Sirt3 in model cells. Meanwhile, the p38 activator U-46619 was used to examine the connection between Sirt3 and p38. We also examined the level of endothelial associated proteins, including occluding, ZO-1 and claudin-4 by using qRT-PCR and western blot.

**Results:**

Our findings indicated that overexpression of Sirt3 decreased the permeability of model cells and promoted in the growth of endothelial cells. However, the activation of p38 could antagonize the function of Sirt3 in HBMEC cells. Moreover, Our results indicated a positive correlation between Sirt3 and inter-endothelial junction proteins. Importantly, PPAR-γ agonist and inhibitor were utilized to investigate the role of PPAR-γ in Sirt3 mediated cell function. Sirt3 was targeted by PPAR-γ in model cells.

**Conclusions:**

Taken together, this research not only demonstrated PPAR-γ might benefit to the growth of endothelial cell though activating Sirt3 but also indicated its potential value in the treatment for ischemic stroke.

## Background

Ischemic cerebral stroke, the second most common cause of death worldwide, is a major disease that threats human health. Despite some newly developed therapies contribute to its treatment, the recurrence and disability rate are still very high. Ischemia-reperfusion (I/R) has induced the dysfunction of vascular, which is the major factor to cause acute ischemic stroke. Cerebral hypoxia and ischemia leads to the injury of parenchymal and vascular cells [1]. The increased permeability of blood–brain barrier (BBB) has been identified as the manifestations of cerebrovascular endothelial injury [1]. Pathologic cerebral edema, which is known to result from increased BBB permeability, is usually found following the ischemic cerebral stroke and critically impacts on the clinical outcome of the stroke [2]. Therefore, the disruption of BBB has been reported as a hallmark of cerebral stroke.

Growing evidence have demonstrated that the permeability of BBB is a promising research target for various brain diseases and drug development [3, 4]. The BBB is formed primarily by brain microvascular endothelial cells (BMECs) [3]. BMECs typically has formed higher resistance of tight junctions than that of macrovascular cells and exhibited lower rates of paracellular leakage, thus mimicking BBB phenotype more closely [5]. Therefore, we simulated ischemic cerebral stroke pathology in vitro by using human brain microvascular endothelial cells (HBMEC) at present study.

Sirt3 belongs to the sirtuin protein family, which plays an important role in the development of neurological diseases [6]. It has been reported that Sirt3 is a critical regulator in cellular protection, which contributes to neurovascular recovery in ischemic stroke [7, 8]. Previous studies have demonstrated that Sirt3 is downregulated in infarct region of ischemic stroke. Moreover, overexpression of Sirt3 protects against glucose deprivation insult though regulating the AMPK-mTOR pathway [9]. In addition, the activity of AKT12 is positively regulated by Sirt3, which is a key factor in neurogenesis and angiogenesis [10]. Recently, it has been confirmed that Sirt1-Sirt3 axis might act as an important modulator in the physiology of BBB, which is a promising therapeutic target for ischemic stroke [11]. This study showed Sirt3 knockdown increased BBB permeability, suggesting Sirt3 is beneficial to neurovascular and functional recovery following chronic ischemic stroke [7, 12]. However, the detailed biological function of Sirt3 is still not fully understood in ischemic stroke and its molecule network still needs to be further investigated.

Peroxisome proliferator-activated receptor-gamma (PPAR-γ) is a ligand-activated nuclear hormone receptor, which is located on human chromosome 3. PPAR--γ level was found increased in ischemic neurons [13]. PPAR-γ plays an important role in the cerebrovascular endothelium of stroke [14, 15]. Growing evidences has indicated that PPAR-γ appears to be a potential therapeutic target due to its anti-inflammatory, anti-apoptosis activities, and anti-ox-dant [15–17]. Moreover, previous report has indicated that knockdown of PPAR-γ leads to bigger infarct [18]. In addition, PPAR-γ suppresses the ischemic neuronal death through inhibiting p22phox and the subsequent NOX activation [15]. Meanwhile, PPAR-γ pathway has the protective role for human ischemic stroke [19]. Recent evidence has suggested Sirt3 prevents the trans-differentiation of cardiac fibroblasts by upregulating PPAR-γ [20]. Although both Sirt3 and PPAR-γ are considered as attractive targets for therapies against stroke and other neurological disorders [7, 8], the full detailed correlation between Sirt3 and PPAR-γ in brain I/R and stroke is still not clear.

It was showed that targeting Sirt3 and PPAR-γ expression in the recovery phase after cerebral ischemia may promote chronic stroke recovery through activation of focal neurogenesis and angiogenesis and prevent post-ischemic inflammation and neuronal damage through regulation of a few critical pathways altered during I/R [7, 21, 22]. The aim of this study was to explore the underling molecular mechanism of the protective roles of Sirt3 and PPAR-γ in I/R models. Human brain microvascular endothelial cells (HBMEC) model is one of the best representative cell models of the human brain endothelium compared to many of other cell lines currently used to study the BBB and thus was selected as our objective in order to further investigate the downstream pathway and mechanism during I/R development. Lentivirus vector was used to induce overexpression of Sirt3, and a few compound inhibitors and agonists of Sirt3, PPAR-γ and p38 MAPK were applied in HBMEC cells. Here, we first reported PPAR-γ as a novel mediator of Sirt3 that together inhibit endothelial cell permeability and ischemic stroke.

## Methods

### Cell culture and ischemia model

Cultured human brain microvascular endothelial cell line (HBMEC) have been authenticated and could be used to construct ischemic cerebral stroke model in vitro [23]. In the present study, HBMEC was purchased from ScienCell (#1000, ScienCell, USA) at Apr. 2018 and cultured in ECM culture solution with 5% FBS, 1% P/S Solution and 1% ECGS in an incubator (37 °C, 5% CO2). The cells were randomly assigned into six groups, including normal HBMECs (BLANK group), HBMECs treated with Sirt3 inhibitor (3-TYP group), HBMECs underwent I/R treatment (details described below) and no plasmid transfection (I/R Control or Control group), I/R treated HBMECs transfected with empty vector plasmid (Vector group), I/R treated HBMECs with overexpressed Sirt3 (Sirt3 group); I/R treated HBMECs overexpressed Sirt3 and cultured with p38 MAPK activator U-46619 (Sirt3 + U-46619 group). The HBMEC cells in each group were seeded in either 96 well plates for cell proliferation assay or in 24-well plates for the rest experiments in a consistent density (4 × 10^4^ cells/ml).

For ischemia/reperfusion model (I/R-model) was simulated by using oxygen and glucose deprivation (OGD) in HBMEC cells according to the method of a previous report [24]. The cell were passaged for 48 h, exposed to sugar-free Kreb’s solution (119 mmol/L NaCl, 4.7 mmol/L KCl, 1.2 mmol/L KH_2_PO_4_, 2 5 mmol/L NaHCO_3_, 2.5 mmol/L CaCl_2_, 1 mmol/L MgCl_2_, pH 7.2), and incubated for 8 h in a hypoxic incubator (1% O_2_, 5% CO_2_, 94% N_2_) at 37 °C. was constructed by replacing media with Krebs buffer containing 5 mM glucose and exposed at ambient air for re-oxygenation. The culture media and cells were simultaneously harvested at 6, 12 and 24 h of recovery. Western blot was used to examine the protein level of Sirt3 and p-p38.

### Overexpression of Sirt3

The pLVX-Puro was utilized to overexpress Sirt3. The full length of Sirt3 cDNA (No.AF083108.2) was inserted into the pLVX-Puro vector by digesting with EcoR I and BamH I. The primer sequences were follows: forward, 5′ –CG GAAT TCATG GCGTTC TGGGGTTG-3′(EcoRI); reverse, 5′-CGGGATCCCTATTTGTCTGGTCCATCAAGC-3′ (BamH I). All plasmids were validated by sequencing. To generate the recombinant Lentivirus Sirt3, 293 T cells were co-transfected with the pLVX-Puro-Sirt3 (1000 ng)、psPAX2(100 ng)、pMD2G(900 ng). A mock lentiviral vector (vector) was served as BLANK control.

### Luciferase reporter assays

Sirt3 gene (NM_001017524) promoter was inserted into the pGL3-Enhancer vector carrying the firefly fluorescent gene (luc2) and pRL-TK with the Renilla fluorescent gene (hRluc). The primer sequences were listed as follows: forward, 5′ –CCCTCGAGACGG CGGAAGTGGTTG-3′ (Xho I); reverse, 5′-CCAAGCTTTCCCTGCCGCCAAG-3′ (Hind III).

The suspension of HBMEC cells cultured in 24 well plates were collected by 0.25% trypsin digestion and pelleted by centrifuging at 1000×g for 5 min. Then, cells were seeded in 6-well plates (5 × 10^5^ cells) per well. After 24 h, the following transfections were performed according to the lip2000 instructions. Each concentration was examined in triplicate. Luciferase activities were measured at 48 h after post-transfection by using Promega Dual-Luciferase Reporter Assay System (E1910) according to the protocol of the manufacture. Firefly luciferase activity was used to normalize the luciferase activity of Renilla.

### Cell proliferation assay

Cell proliferation assay was measured by using a Cell Counting Kit-8 (CCK8) according to the instructions of the manufacturer. Cells were seeded onto 96-well plates (2 × 10^3^ cells/well), and cell proliferation was documented at 0, 24, 48, 72 h respectively. OD450 values were examined by DNM-9602 microplate reader (Pulang New Technology Corp. Beijing).

### FITC-dextran and TEER

The analysis of cell permeability was followed by the previous method [25]. In brief, the HBMEC cells were seeded in the upper chamber of 24-well Transwell plates (2 × 10^4^ cells/well), and 100 μl and 600 μl of the medium were added to the upper and lower chambers respectively. The cells were treated for 24 h in the experimental group, and 1 mg/ml fluorescein isothiocyanate (FITC)-labelled dextrans was added at 0 and 24 h and incubate for 2 h. Then, the fluorescence intensity of FITC was measured by a microplate reader (excitation wavelength 490 nm, emission wavelength 520 nm), and the permeability was calculated according to a standard curve. After 24 h, the Trans-epithelial/endothelial electrical resistance (TEER) of the cells was measured by Millicell ERS-2 Voltohmmeter (Millipore, Bedfoed, MA). Moreover, blank holes (without cells) were set and the resistance value was measured.

### Cell apoptosis

Cell apoptosis profile was analyzed by Flow cytometry. The HBMEC cells in each group were seeded in 24-well plates (2 × 10^4^ cells/well), were digested by trypsin. Then, re-suspended and incubated in phosphate-buffered saline containing Annexin V-FITC and propidium iodide (PI). Accuri C6 flow cytometer (BD biosciences, CA, USA) was used to analyze cells according to the instruction of the manufacture.

### Western blot analysis

Total protein was extracted by the method as described previously [26] and BCA assay was used to determine the protein content. Protein were separated by gradient SDS/PAGE gels and subsequently transferred to PVDF membranes. The membranes were blocked by 5% skim milk for 1 h and incubated overnight at 4 °C with primary antibodies respectively, including Sirt3 (1:1000), p38 (1: 1000), p-p38 (1:1000), Occludin(1: 1000), ZO-1(1:1000), Claudin-4 (1:1000), GAPDH (1: 1000). After that, the membrane was washed with TBST for 3 times and incubated with HRP-conjugated secondary antibodies (1:1000) for 1 h, at 37 °C. GAPDH was used as an internal control. The enhanced chemiluminescence (ECL) system (Tanon, Shanghai, China) was used to detect the protein content.

### Real time PCR

Briefly, the total RNA of different samples was extracted by using Trizol reagent (invitrogen). Then, a qRT-PCR SYBR Green Kit (Thermo) was used to reverse transcribe the total RNA into cDNA according to manufacturer’s instructions. Real time PCR was performed by using quantitative PCR in the presence of a fluorescent dye (SYBR Green I). GAPDH was used to normalize the gene expression. The relative gene expression was calculated using the 2^*-ΔΔCt*^ method. The primers that used in this study were listed as follows: GAPDH: F: 5′ AATCCCATCACCATCTTC 3′, R: 5′ AGGCTGTTGTCATACTTC 3′; Sirt3: F: 5′ CCTTGGCTTGGCATCCTC 3′, R: 5′ GCACAAGGTCCCGCATCTC 3′; claudin 4: F: 5′ TGGGGCTACAGGTAATGG 3′, R: 5′ ATGATGCTGATGATGACGAG 3′; zona occludens 1: F: 5′ TTGGCGAGAAACGCTATG 3′, R: 5′ TCTGAGATGGAGGTGGGTC 3′; occluding: F: 5′ CCCATCTGACTATGTGGAAAG 3′, R: 5′ CACCGCTGCTGTAACGAG 3′.

### Statistical analysis

All data are presented as the mean ± SD. Data were analyzed by using one-way ANOVA followed by the Student’s t-test for unpaired data with Bonferroni correction. Square roots of tissue cell counts were compared using one-way ANOVA. Statistical significace was accepted by *p*-value < 0.05.

## Results

### Sirt3 was downregulated in HBMEC cells after I/R

**Fig. 1:**
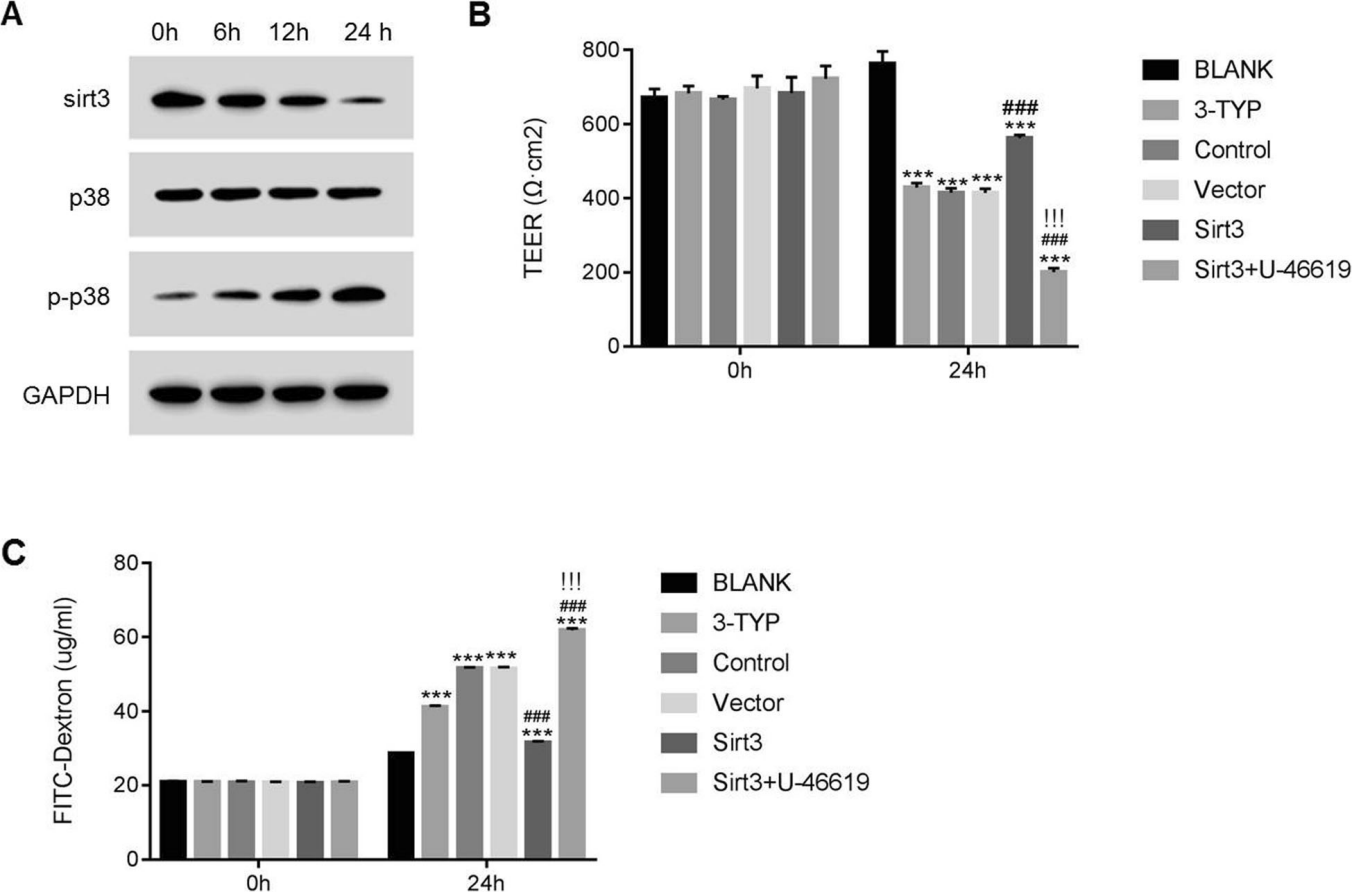
Sirt3 attenuates endothelial cell permeability in p38 dependent manner. **a**, Protein levels of Sirt3 and p38 during early post-reperfusion stages (6 h–24 h) of HBMECs I/R injury model, as compared to control groups with no reperfusion treatment (0 h). B & C, TEER measurement (**b**) and dextran permeability testing (**c**) in HBMEC cells either from normal HBMECs with or without SIRT3 inhibited by Sirtuin inhibitor 3-TYP (BLANK and 3-TYP), or from HBMECs at 24 h of reperfusion of I/R injury with Sirt3 overexpression, together with or without treatment of a specific p38 MAP kinase the activator U-46619 (Control, Vector, Sirt3 and Sirt3 + U-46619). The column graph are representative data of an *n* = 3 performed in quadruplicate. ***, *p* < 0.001 as compared to the normal HBMECs model (BLANK). ^###^, p < 0.001 as compared to the non-overexpression control group (Control) and^!!!^ as compared to the Sirt3 overexpression group (Sirt3). TEER: Transepithelial/endothelial Electrical Resistance

Western blot was used to examine the protein level of Sirt3, p38 and p-p38 in HBMEC cells after I/R treatment. As shown in Fig. 1a, we found that the level of Sirt3 was downregulated in HBMEC cells after I/R treatment. Meanwhile, the treatment of I/R promoted the level of p-p38 in HBMEC cells.

### The TEER value was positively correlated with the level of Sirt3 in HBMEC cells

Next, we investigated the function of Sirt3 in the process of TEER in I/R model cells. A Sirt3 inhibitor 3-TYP was used to silence the expression of Sirt3. As shown in Fig. 1b, the TEER value was significantly reduced from 750 Ω.cm^2^ to 400 Ω.cm^2^ in 3-TYP treated cells (3-TYP vs. BLANK, *p* < 0.001). Moreover, the treatment of I/R also contributed to inhibit the activity of TEER in similar pattern (Control vs. BLANK, *p* < 0.001).

Importantly, overexpression of Sirt3 significantly increased the level of TEER in I/R model cells from 400 Ω.cm^2^ to 550 Ω.cm^2^ (Sirt3 vs. Control or Vector, p < 0.001). However, this effect was deeply abolished by the p38 MAPK activator U-46619 from 550 Ω.cm^2^ to 200 Ω.cm^2^ (Sirt3 + U-46619 vs. Sirt3, p < 0.001). Taken together, Sirt3 was a positive mediator in the regulation of TEER process. Moreover, Sirt3 and p38 might involve in the same pathway. In addition, we also examined the permeability of different cells as indicated in Fig. 1b.

As shown in Fig. 1c, the Sirt3 inhibitor 3-TYP significantly promoted the permeability of HBMEC cells, with the FITC signal increased from 25 μg/ml to 40 μg/ml (3-TYP vs. BLANK, *p* < 0.001). Moreover, the permeability of HBMEC cells was also significantly upregulated by I/R treatment (Control vs. BLANK, *p* < 0.001). Moreover, overexpression of Sirt3 significantly suppressed the permeability of I/R cells from 50 μg/ml to 25 μg/ml (Sirt3 vs. Control or Vector, p < 0.001). Interestingly, the activator U-46619 remarkably disrupted this effect, restoring the FITC sign from 25 μg/ml to over 60 μg/ml (Sirt3 + U-46619 vs. Sirt3, *p* < 0.001). These results further indicated that p38 antagonized the function of Sirt3 in HBMEC cells.

### Sirt3 promotes the growth of endothelial cells

**Fig. 2:**
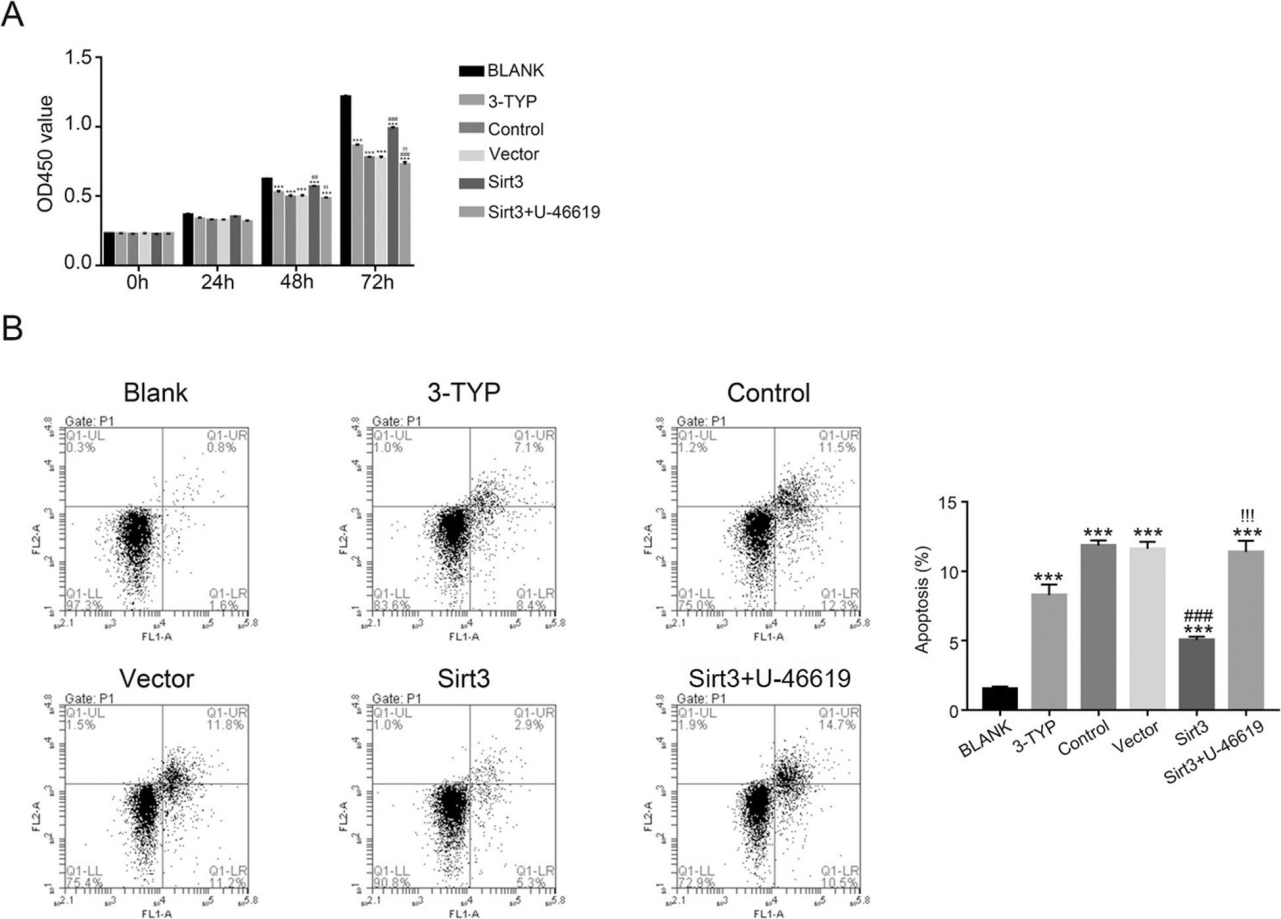
Sirt3 promotes endothelial cell proliferation and inhibits cell apoptosis. **a**, Cell growth curves of endothelial cells were generated using CCK8 during post-reperfusion stages (24–72 h), as compared to control groups with no reperfusion treatment (0 h). **b**, Cell apoptosis analysis of cells at 24 h of reperfusion after I/R injury. Cells were either from normal HBMECs with SIRT3 inhibition (BLANK and 3-TYP), or from I/R injury HBMECs at 24 h of post-reperfusion with SIRT3 overexpression, together with or without U-46619 treatment (Control, Vector, Sirt3 and Sirt3 + U-46619). ***, *p* < 0.001 as compared to the normal HBMECs model (BLANK). ^###^, p < 0.001 as compared to the non-overexpression control group (Control) and^!!!^ as compared to the Sirt3 overexpression group (Sirt3)

Next, we also examined the function of Sirt3 in the regulation of cell growth. As shown in Fig. 2a, the cell proliferation rate was significantly reduced in the present of 3-TYP treated cells with OD value at 72 h post-seeding dropped from 1.2 to 0.8 (3-TYP vs. BLANK, *p* < 0.001). Moreover, I/R treatment also significantly inhibited the proliferation rate of endothelial cells with OD value at 72 h post-seeding dropped from 1.2 to 0.7 (Control vs. BLANK, *p* < 0.001). Meanwhile, overexpression of Sirt3 significantly promoted the proliferation rate of endothelial cells (Sirt3 vs. Control or Vector, *p* < 0.001). However, this effect was also deeply suppressed by the p38 and p38 MAPK activator U-46619 with OD value at 72 h timepoint increased dropped more than one quarter (Sirt3 + U-46619 vs. Sirt3, *p* < 0.001).

Moreover, we also examined the apoptosis profile of different cells as indicated (Fig. 2b). In consistent to the cell proliferation results, the Sirt3 inhibitor 3-TYP and I/R treatment significantly accelerated the apoptosis of endothelial cells (from less than 2 to 8% and 13%, both *p* < 0.001). Obviously, Fig. 2b showed overexpression of Sirt3 significantly decreased the apoptosis ratio of endothelial cells (from 13 to 5%, p < 0.001), which was significantly restored by the p38 MAPK activator U-46619 (from 5 to 13%, p < 0.001).

### Suppressing the expression of Sirts inhibits the level of inter-endothelial junction proteins

Inter endothelial junction proteins plays a key role in the permeability of BBB para-cellular. In this study, inhibiting the expression of Sirt3 and I/R treatment in endothelial cells affected the function barrier. To further investigated the correlation between Sirt3 and inter-endothelial junction proteins, qRT-PCR and western blot were used to quantify the level of inter-endothelial junction proteins, including occludin, ZO-1 and Claudin-4.

**Fig. 3:**
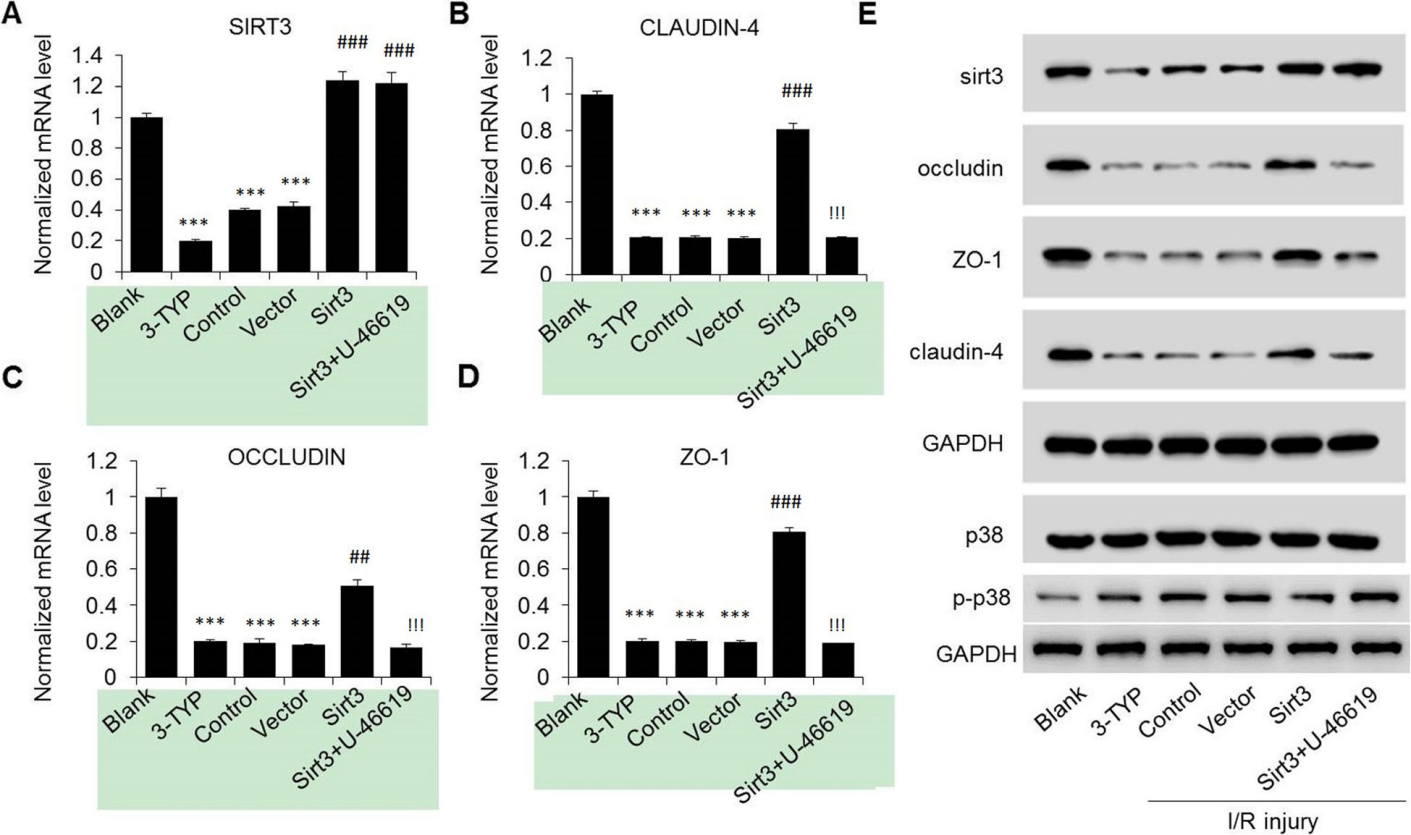
Expression of endothelial associated protein as induced in I/R model mRNA (**a**-**d**) and protein levels (**e**) of Sirt3, p38 as well as a few other permeability associate proteins in HBMEC at 24 h of reperfusion after I/R injury. Cells were either from normal HBMECs with SIRT3 inhibition (BLANK and 3-TYP), or from I/R injury HBMECs at 24 h of post-reperfusion with SIRT3 overexpression, together with or without U-46619 treatment (Control, Vector, Sirt3 and Sirt3 + U-46619). ***, p < 0.001 as compared to the normal HBMECs model (BLANK). ^###^ or ^##^, p < 0.001 or *p* < 0.01 as compared to the non-overexpression control group (Control) and^!!!^ as compared to the Sirt3 overexpression group (Sirt3)

As shown in Fig. 3a-d, the mRNA levels of occludin, ZO-1 and Claudin-4 were significantly suppressed in the inhibitor 3-TYP and I/R treated cells (down from 1.0 to 0.2, *p* < 0.001). Meanwhile, overexpression of Sirt3 significantly upregulated the mRNA level of occludin, ZO-1 and Claudin-4 (up from *p* < 0.01, p < 0.001, p < 0.001 respectively), which was significantly restored by the p38 the activator U-46619 (all p < 0.001). As for protein content, we also obtained the similar results (Fig. 3e). Therefore, Sirt3 was a positive mediator in the regulation of inter-endothelial junction proteins. The function of Sirt3 in the regulation of permeability and growth of endothelial cells might achieve though regulating inter-endothelial junction proteins in I/R model.

Moreover, the p38 MAPK activator U-46619 significantly reverse Sirt3 expression and Sirt3 mediated regulation in I/R model (Figs. 1, 2 and 3a-d). Overexpression of Sirt3 highly inhibited the phosphorylation of p38 (Fig. 3e). Taken together, these results indicate that p38 involved in the molecule network of Sirt3 in I/R model.

### PPAR-γ contributed to the expression of Sirt3 in endothelial cells

**Fig. 4:**
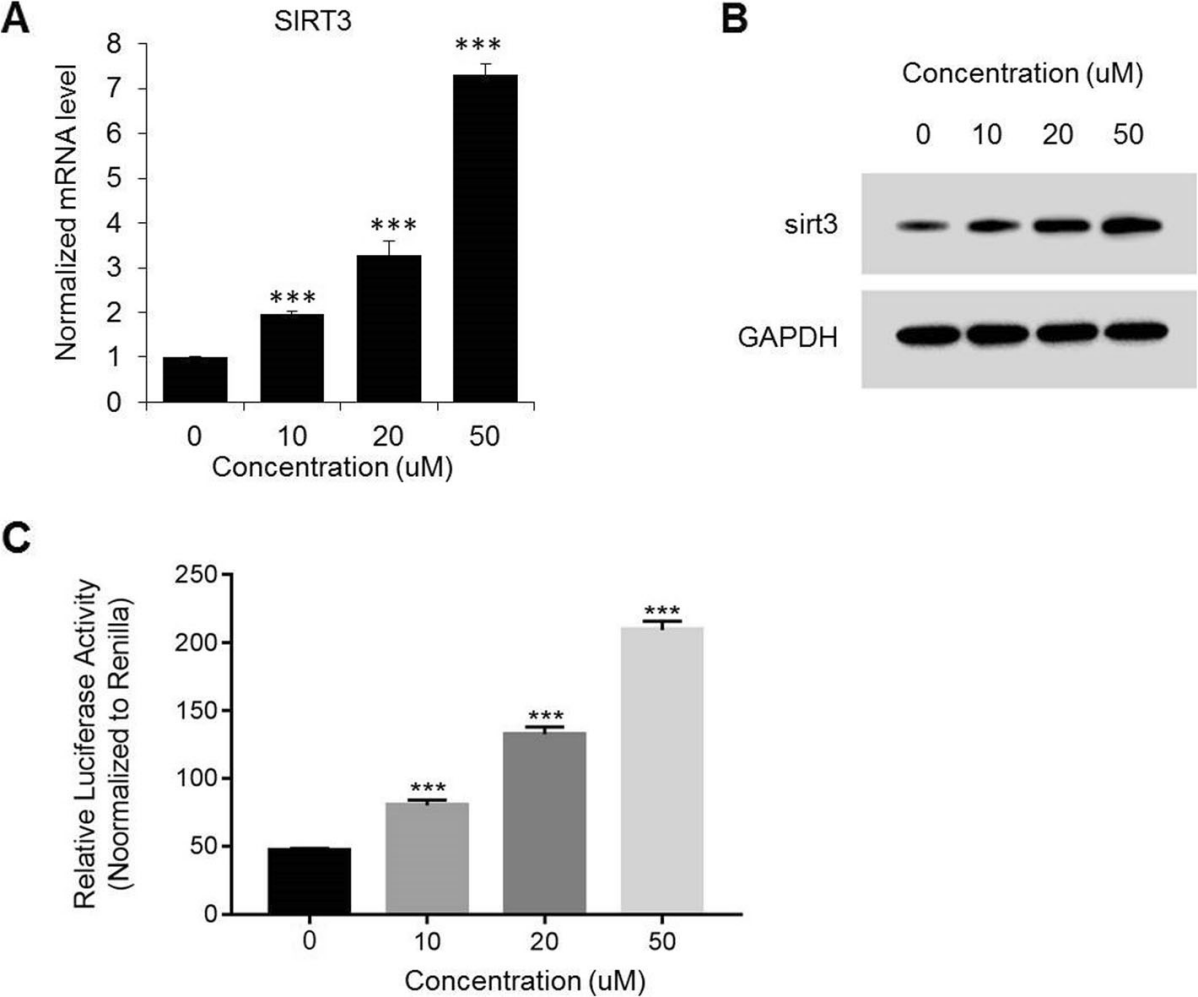
PPAR-γ agonist promotes Sirt3 expression and activation. A, mRNA (**a**) and protein levels (**b**) of Sirt3 after PPAR-γ agonist treatment (in doses of 10, 20, and 50 uM and compared to non-treatment control) at 24 h of reperfusion of I/R injury HBMECs. **c**, Luciferase assays showed the activation of SIRT3 promoter region after PPAR-γ agonist treatment (in doses of 10, 20, and 50 uM and compared to non-treatment control) at 24 h of reperfusion of I/R injury HBMECs. ***, p < 0.001 comparing individual column to the column on the left

We also evaluated the level of Sirt3 in endothelial cells that treated by PPAR-γ agonists with different doses, including 10, 20 and 50 uM, as compared to control (0 uM). With the concentration of PPAR-γ agonists rising, both the mRNA and protein level of Sirt3 were gradually upregulated in endothelial cells with up to 7-fold mRNA increase at the 50 uM treatment group comparing to non-treatment control (Fig. 4a-b, all *p* < 0.001). Moreover, luciferase reporter assay was performed to examine the luciferase activity of Sirt3 in different cells as indicated. Our results indicated that the luciferase activity of Sirt3 was enhanced by PPAR-γ agonists as more than 4-fold increase was showed at the 50 uM treatment group comparing to control (Fig. 4c, all *p* < 0.001).

**Fig. 5:**
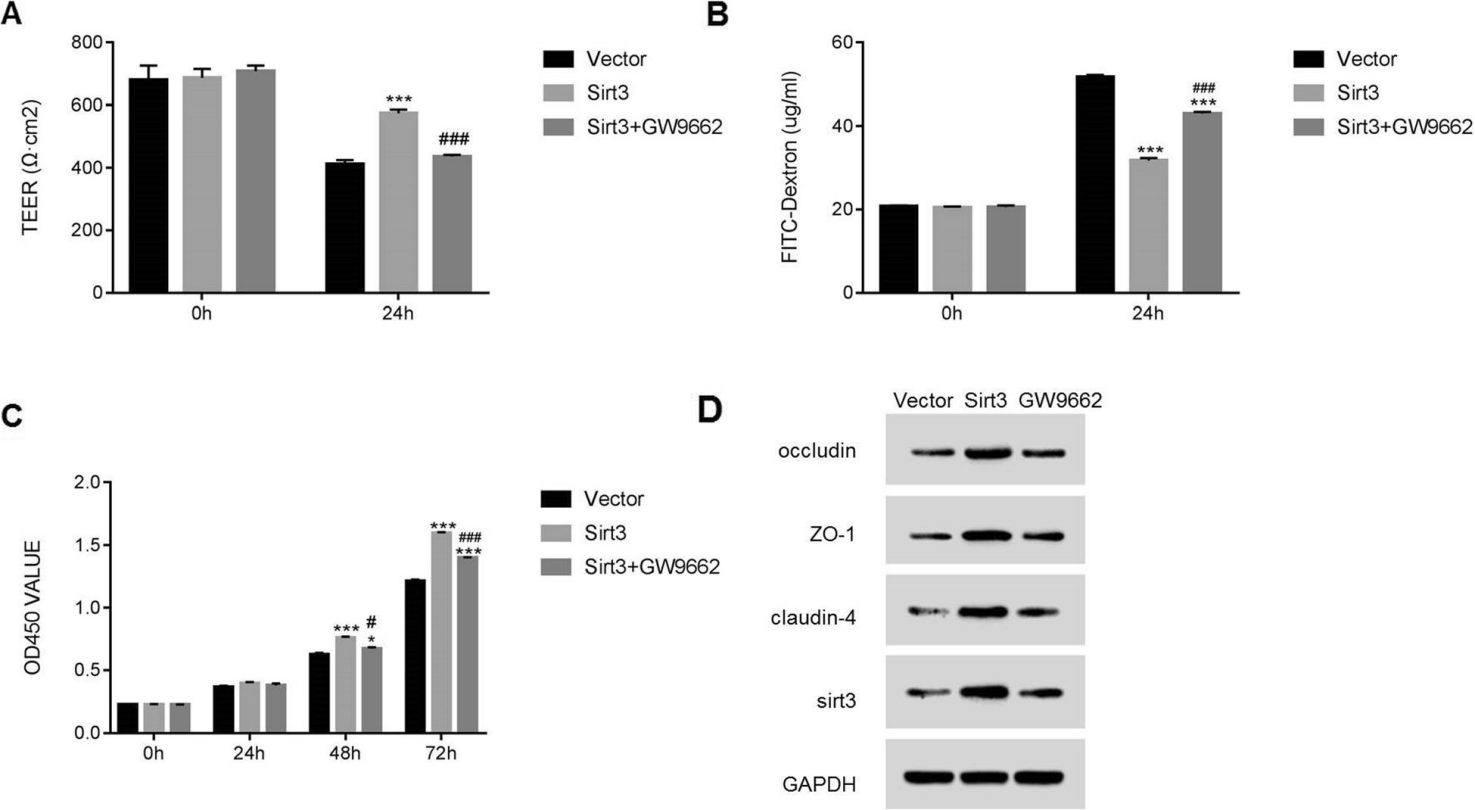
PPAR- γ inhibitor reverses Sirt3 mediated cell function in HBMEC cells. A-C, TEER measurement (**a**) and dextran permeability testing (**b**) as well as CCK-8 cell proliferation assay (**c**) and western blot (**d**) were performed in HBMEC cells to identify the roles of PPAR- γ inhibitor. (**e**). ***, p < 0.001 as compared to the empty vector group (Vector). ^#^, ^###^, *p* < 0.05 or p < 0.001 as compared to the Sirt3 overexpression group (Sirt3)

To further examine the correlation between Sirt3 and PPAR-γ agonists in endothelial cells, a specific PPAR-γ inhibitor GW9662 was used to culture I/R cells. As shown in Fig. 5a, the TEER value was significantly reduced in I/R cells (Vector groups in 0 h vs. 24 h treatment, p < 0.001), increased by Sirt3 (Sirt3 vs. Vector, p < 0.001) and restored by PPAR-γ inhibition (Sirt3 + GW9662 vs. Sirt3, p < 0.001). Moreover, the inhibitor GW9662 improved the permeability of I/R cells as it was reduced by Sirt3 (both p < 0.001, Fig. 5b). In addition, Sirt3 mediated increase of cell proliferation was significantly suppressed by GW9662 (p < 0.001, Fig. 5c). Besides that, we also examined the protein level of occludin, ZO-1 and Claudin-4 in GW9662 cultured cells. As shown in Fig. 5d, the protein level of occludin, ZO-1 and Claudin-4 were also inhibited by the inhibitor GW9662 (Fig. 5d). Taken together, these results indicated that PPAR-γ might reduce the permeability of BBB though promoting Sirt3 and subsequently upregulating the expression of the intercellular connective proteins.

## Discussion

Sirt3 has played a key role in a variety of cellular processes, which has a protective effect in myocardial ischemia and neurodegenerative diseases. BBB can effectively protect nerve cells from many harmful substances. Previous report has indicated that BBB is identified as a common chemical damage marker, which is associated with the upregulation of brain vascular endothelium permeability [27]. Furthermore, it has been confirmed that Sirt3 has upregulated the expression of PPAR-γ [20] However, it is still unclear the biological function of PPAR-γ in BBB. Here our results demonstrated that overexpression of Sirt3 may reduce the permeability of endothelial cell through inhibiting the phosphorylation of p38 and promoting BBB cell-permeability-associated proteins, including occludin, ZO-1, and claudin-4. Meanwhile, Sirt3 was a pro-proliferation factor and anti-apoptosis of HBMEC cells. We also obtained the similar results in cells that treated by PPAR-γ inhibitors on these events. In conclusion, our results indicate that PPAR-γ may improve endothelial cell permeability and ischemic stroke through inhibiting the expression of Sirt3.

As a member of the sirtuins family, Sirt3 is highly expressed in brain and other nervous system tissues [28]. Our results showed that the level of Sirt3 and p-p38 were increased in cells with the treatment of I/R. Moreover, cerebral hypoxia and ischemia increase the permeability of BBB and reduced the expression of inter-endothelial junction proteins, including VE-Cadherin, claudin-5 and ZO-1. In addition, the expressions of cell tight junction protein were inhibited by Sirt3 inhibitor and I/R treatment, which subsequently increased the permeability of endothelial cells and its apoptosis rate. Meanwhile, overexpression of Sirt3 promoted the expression of occludin, ZO-1 and Claudin-4 in I/R model, which decreased the permeability of BBB. Moreover, our results demonstrated that Sirt3 was a pro-proliferation and anti-apoptosis regulator and positively correlated with p-p38.

Activation of MAPK, including p38 and Erk1/2, has been reported to be involved in the damage of I/R after transient middle cerebral occlusion (tMACO) [29]. Claudin, occludin and ZO-1 are essential molecules that involved in BBB structural tight junctions (TJs) [30]. Moreover, previous data has indicated that H_2_O_2_ stimulation causes sustained high levels of p38 phosphorylation, increasing cell permeability of FITC-dextran [31]. In this study, our results indicated that the effects of Sirt3 were abolished by p38 MAPK inhibitors in I/R cells.

The Sirt family is involved in the regulation of many neurological diseases, but the biological function of Sirt in the permeability of endothelial cells has not been fully established. Gao et al. [32] have demonstrated that overexpression of Sirt1 may regulate the proliferation and migration of endothelial cells, which indicated the key role of Sirt1 in angiogenesis. Moreover, Sirt1 is upregulated by sphingosine kinase 1 (SPHK1) and S1P in human umbilical vein endothelial cells through multiple pathways, including p38 MAPK, ERK and AKT signals. However, the function of Sirt3 in the activation of p38 MAPK signal is still unclear in I/R model. Our results indicated that overexpression of Sirt3 induced the phosphorylation of p38 and reduce the permeability of BBB though regulating the expression of intercellular connexin in endothelial cells and further promoted its growth. Furthermore, our data provided evidence therapy targeting Sirt3-p38 MAPK pathway might be useful for impairment of functional recovery following chronic ischemic stroke.

Growing evidences have demonstrated that PPAR-γ activators significantly reduced the volume of cerebral infarction, improve blood-brain barrier permeability and reduce apoptosis [33, 34], but the specific mechanism has not been determined. It has been confirmed that ischemic stroke involves PPAR-γ signaling [33]. Recently, Wu et al. [34] found that *C. nutans* extract inhibits apoptosis process by increasing the activity of PPAR-γ signaling pathway. The preventive and therapeutic effects of *C. nutans* on ischemic stroke are identified. Although a few studies have mentioned the effect of Sirt3 on PPAR-γ [20], little is known about the role of PPAR-γ in the I/R effect on the Sirt family. Our study is the first report that indicates Sirt3 might be the downstream target and a novel aspect explaining the beneficial and clinically relevant PPAR-γ effectively in improving neurodegenerative and inflammatory processes during stroke. In this research, our results found that PPAR-γ induces the up-regulation of Sirt3 and reduce the permeability of BBB though promoting the expression of tight junction proteins occludin, including ZO-1 and Claudin-4. Currently, new compounds or other mediators of SIRT3 and PPAR-γ have constituted productive research directions. Mediators of Sirt3 includes Traditional Chinese medicine (Resveratrol, Polydatin, Berberine etc.), small molecule activators (Melatonin, Adjudin, Minocycline) and triggers of other signaling pathways (EphB2 signaling, cAMP/PKA signaling and Sirt1 signaling) [12, 35]. Similarly, a few compounds such as thiazolidinediones (TZDs), icosinoids-like leukotriene B4 and 8(S)-hydroxy-eicosatetraenoic acid have emerged as potent, exogenous agonists of PPAR and are being prescribed for diseases [36, 37]. We believe that studies on SIRT3 and PPAR-γ will soon generate new approaches for the treatment of stroke.

## Conclusion

In summary, we present an interesting mechanism that reveals new therapeutic targets for PPAR-γ and Sirt3 for ischemic stroke and provided new ideas for further research. However, this study was mostly performed in in vitro studies that using cell cultures as model system to recreate consequences of ischemic stroke. More extended investigation in in vivo models such as animal models is therefore needed to confirm the effect of targeting SIRT3 and PPAR-γ in stroke, especially for the findings are not in agreement between different in vitro models.

## Data Availability

All data generated or analyzed during this study are included in this published article.

## Abbreviations

BBB: Blood–brain barrier
BMECs: Brain microvascular endothelial cells
CCK8: Cell Counting Kit-8
FITC: Fluorescein isothiocyanate
HBMEC: Human brain microvascular endothelial cells
I/R: Ischemia-reperfusion
OD: Optical density
PI: Propidium iodide
PPAR-γ: Peroxisome proliferator-activated receptor-gamma
SPHK1: Sphingosine kinase 1
TEER: Trans-epithelial/endothelial electrical resistance
tMACO: transient middle cerebral occlusion
